# Unlocking the post-transplant microenvironment for successful islet function and survival

**DOI:** 10.3389/fendo.2023.1250126

**Published:** 2023-08-29

**Authors:** Daniel T. Doherty, Hussein A. Khambalia, David van Dellen, Rachel E. Jennings, Karen Piper Hanley

**Affiliations:** ^1^ Faculty of Biology, Medicine & Health, University of Manchester, Manchester, United Kingdom; ^2^ Department of Renal & Pancreatic Transplantation, Manchester University NHS Foundation Trust, Manchester, United Kingdom; ^3^ Department of Endocrinology, Manchester University NHS Foundation Trust, Manchester, United Kingdom

**Keywords:** islet transplantation, extra-cellular matrix (ECM), tissue response, beta-cell replacement, T1DM (type 1 diabetes mellitus)

## Abstract

Islet transplantation (IT) offers the potential to restore euglycemia for patients with type 1 diabetes mellitus (T1DM). Despite improvements in islet isolation techniques and immunosuppressive regimes, outcomes remain suboptimal with UK five-year graft survivals (5YGS) of 55% and most patients still requiring exogenous insulin after multiple islet infusions. Native islets have a significant non-endocrine component with dense extra-cellular matrix (ECM), important for islet development, cell survival and function. Collagenase isolation necessarily disrupts this complex islet microenvironment, leaving islets devoid of a supporting framework and increasing vulnerability of transplanted islets. Following portal venous transplantation, a liver injury response is potentially induced, which typically results in inflammation and ECM deposition from liver specific myofibroblasts. The impact of this response may have important impact on islet survival and function. A fibroblast response and ECM deposition at the kidney capsule and eye chamber alongside other implantation sites have been shown to be beneficial for survival and function. Investigating the implantation site microenvironment and the interactions of transplanted islets with ECM proteins may reveal therapeutic interventions to improve IT and stem-cell derived beta-cell therapy.

## Type 1 diabetes mellitus and beta-cell replacement

A patient with T1DM administers 65,000 injections and checks their blood glucose levels 80,000 times during their lifetime (1). T1DM results from autoimmune destruction of beta-cells in the pancreatic Islets of Langerhans, preventing insulin production. T1DM affects approximately 400,000 people in the UK, 29,000 of whom are children with a predicted rise to 600,000 and 48,000 respectively in 2035 (2). Worldwide this figure is approximately 37 million (3). Microvascular and macrovascular complications are characteristic of T1DM manifested clinically with retinopathy, end stage renal failure, stroke and cardiovascular disease the hallmarks (4–6).

Beta-cell replacement strategies seek to restore insulin production capabilities thereby reducing exogenous insulin requirements. Pancreas transplant (PT) outcomes have steadily improved (7), but devastating complications with associated morbidity and mortality (8–10). Transplantation of pancreatic islets to reinstate pancreatic endocrine function without the complications produced by exocrine components and free of vascular and enteric anastomoses poses an attractive alternative. Infusion of isolated islets into the hepatic parenchyma via the portal vein offers this potential (11). However, islet supply is limited and multiple transplants are needed due to initial beta-cell loss, progressive functional decline to augment graft longevity (12). This approach was revolutionised using a steroid free immunosuppression regime and increased delivery of islet mass in 2000, termed the Edmonton Protocol, demonstrating the first reported insulin independence (11). Islet transplantation (IT) remains the most commonly clinically available cellular therapy for T1DM, although stem-cell derived islets (SCI) strategies are emerging (**Table 1**). These obviate the limitations of donor supply and have the potential to become a considered treatment for patients with T1DM (17, 19). Exciting progress has been made through clinical trials. VC-02 has assessed endocrine progenitor (PEC-01) cells in combination with an encapsulation device with positive serum C-peptides in some recipients (17, 18). Early results from VX-880, have demonstrated insulin independence after SCI portal venous infusion in a small number of patients (20, 21) and VCTX210 which uses CRISPR mediated gene editing to reduce immunogenicity is ongoing (22–24). Challenges remain, including concerns for off target growth, which has not been seen in these studies and the optimal differentiation strategy.

**Table 1:** Therapeutic options for type 1 diabetes mellitus.

Insulin Delivery	Treatment	Challenges	Notable Studies
Exogenous Insulin	Subcutaneous Injection	Tolerance/adherence Challenging with complex control Hypoglycaemia	Banting et al 1922 (13)
	Automated Devices	Device tolerance Hypoglycaemia Variable availability/cost implications	Pickup & Sutton 2008 (14)
Beta-Cell Replacement	Pancreas Transplant	Finite resource Major surgery	Gruessner & Gruessner 2022 (15)
	Islet Cell Transplant	Finite resource Functional decline	Shapiro et al 2000 (11) Marfil-Garza et al 2022 (16)
	Stem-Cell Derived Islets	Off-target growth Limited data Differentiation optimisation	Shapiro et al 2021 (17) Ramzy et al 2021 (18)

## Characteristics & outcomes of islet transplantation

IT is employed to reduce the burden of T1DM and is indicated in the UK for patients with T1DM suffering from episodes of hypoglycaemic unawareness or extremely labile glucose control (5). In the UK, the aim of IT is to improve glycaemic control with consequent reduction in disease burden, rather than achieving insulin independence. Between 2011 and 2020, approximately 30 ITs were performed annually in the UK. The majority (approximately 90%) from donors after brain death. UK islet graft survival is comparable to simultaneous kidney and pancreas transplant at 1 year, but reduced at 5 years (5YGS: 81% vs. 55% respectively); however, direct comparison is challenging due to differences in definitions of PT and IT failure (25).

For UK clinical use, islet preparations must achieve a minimum yield of 250,000 Islet Equivalents (IEQ), with purity of over 50% endocrine tissue, determined by dithizone (DTZ) staining and 70% viability. In addition, for a first transplant in the UK, the yield must be sufficient to achieved 5000 IEQ/Kg for the designated recipient or 10000 IEQ/Kg if to be used for a supplementary islet transplant (26). UK recipients will usually receive repeat transplantation within 6 months to target a total islet mass of 10,000 Islet Equivalents (IEQ)/kg following initial transplantation which has been demonstrated to have improved graft function and 5 year survival (27). This supports findings in small animal models that have demonstrated improved islet allograft survival following a second transplant compared to primary transplantation in mice (28). Parameters of purity, yield and viability for quality control vary internationally with some suggesting 20-30% purity is sufficient and 80% is viability required (12, 29).

Purity in islet preparations is considered a marker of successful islet isolation and predictive of graft success. However, there is evidence to suggest that long term functionality is not determined by endocrine content alone. It has been shown that long term glycaemic control is superior in recipients with islet infusions with <50% purity compared to >50% with similar islet viability and total islet mass (30). In these recipients, higher post-transplant levels of Ca 19-9 and glucagon activity were associated with improved metabolic profile at 5 years after islet transplant suggesting that other endocrine and exocrine pancreatic components may be important, rather than beta-cell mass alone (31–33). In addition, the supplementation of islet preparations with mesenchymal stromal cells can improve islet survival during the isolation process through reduction in islet stress as well as supporting islet survival and functionality post-transplant in rodents (34–36).

Despite the promise of IT, graft longevity remains elusive. Analysis of outcomes of a Swiss-French islet network demonstrated that insulin independence persisted in only 1 of 21 patients at 10 years follow-up, whilst 73% remained free of severe hypoglycaemic episodes (37). Another single centre study of 10 year outcomes revealed 78% graft function at 10 years, although quality of glycaemic control reduced over time (38). Twenty-year follow up in a cohort of 255 islet recipients from the University of Alberta demonstrated median graft survival of 5.9 years with 71% achieving insulin independence, which gradually reduced to 8% after 20 years (16).

The great progress in IT has only accentuated the lack of donor islets and the need to understand why islet loss continues post-transplantation. The mechanism for islet loss cannot be solely attributed to ongoing autoimmunity or allogeneity as loss follows transplantation for other forms of pancreatic disease and in syngeneic models of islet transplantation (39–42). Loss of islet mass is multifactorial, and aetiology varies along the transplantation pathway. Initially there is a large reduction in viable islet mass, with evidence of loss of 50% of the transplanted islet mass (43, 44). Early islet loss is mediated by a combination of mechanical and immunological factors as well as loss driven by ischaemia and islet hypoxia (45). Here, we will discuss the importance of the islet microenvironment, describing the native islet structure, how this niche is destroyed by islet isolation and how islet transplantation triggers implantation tissue injury responses which shapes the post-transplant microenvironment. Examination of this concept will reveal improvements in engraftment of both islet allografts and SCI.

## Islet architecture

### Non-endocrine cells

Non-endocrine cell types are important to islet function; predominantly endothelial cells which are a component of the network of intra-islet microvasculature which is five times denser than the exocrine pancreas alongside dendritic cells and nerve fibres (46). In human islets endocrine cell types are close to vasculature, in a non-patterned distribution along the vessel course. Each islet receives arterial supply from up to five arterioles which form a glomerulus-like network of capillaries within which endocrine cells are sited, closely located to vessels, with a double basement membrane (BM) between beta-cells and capillary endothelial cells, both of which heavily express lutheran glycoprotein (47, 48). Polarity of beta-cells related to vessels is exhibited on electron microscopy of rat islets, demonstrating how cell-matrix interactions with this BM can influence morphology (49). Islet capillaries demonstrate a thinner endothelium with significantly higher levels of fenestration, with these characteristics highly delineated at the islet-exocrine interface (50).

Native islet ECM producing cells are not well characterised, but evidence is emerging for the role of islet stellate cells (ISCs). Similar, but distinct from pancreatic stellate cell population, which are implicated in pancreatic fibrosis and exocrine disease, they possess a quiescent and activated state in which they are thought responsible for excessive ECM deposition and implicated in Type 2 Diabetes Mellitus (T2DM) (51, 52). However, when isolated ISCs are co-cultured with mouse islets, improved insulin production is seen (53). Others have described a pericyte cell type, with similar properties, capable of activation into a myofibroblast subtype. Such cells have actions on insulin production through control of microvasculature and directly and have also been demonstrated to produce ECM components (54, 55). The proposition that overactivity of these tissue resident fibroblasts impairs islet function in T2DM may draw comparison with the post-transplant microenvironment.

### Native islet extra-cellular matrix

The extra-cellular matrix (ECM) is a complex environment, composed of multiple proteins, glycoproteins and proteoglycans. These networks, which are found across tissue types, can bind with cell surface receptors to influence cell differentiation, survival and function. This can be achieved via integrin receptors and through mechano-sensing of transmitted loads through frequency and intensity of cell-ECM interactions. The cytoplasmic tails of integrin receptors bind with the intra-cellular actin cytoskeleton and activate pathways involved with cell survival and function and allow bi-directional communication with the local micro-environment (56).

The importance of the islet ECM has led to the investigation of the impact of predominant BM components on beta-cell activity. Integrin signalling is important for the development of beta-cells, survival, and functional state. The composition of ECM and integrin expression changes through development and adulthood but in both mice and human developing islets, knock outs of integrin types can lead to reduced islet development and functionality (57, 58). Antibody blockade of integrin α_1_ and integrin β_1_ subunits reduce cell adhesion and migration of foetal and adult human islets *in vitro* (59). Culture of foetal islets with collagen IV also promotes insulin function compared to other ECM components and this action is reversed by integrin α_1_β_1_ antibody blockade (59).

Laminins and glycoproteins including fibronectin (FN) are also thought to impact survival and function. FN is a key component of developing islets and activates integrin α_v_β_1_. Blockade with 270 RGD-peptide analogue in immunodeficient mice after renal subcapsular transplant of human foetal pancreas fragments, results in aberrant islet development with loss of conventional structure and a reduction in insulin positive cells (60). Whilst the ability of proteoglycans to bind and regulate availability of growth factors such as fibroblast growth factor, vascular endothelial growth factors (VEGF) and connective tissue growth factor has important implications for beta-cells (61). For example, reduction in expression of VEGF-A in mouse beta-cells causes impaired insulin production with reduction in fenestrations of intra-islet vasculature and reduces revascularisation at 1 month after these cells are transplanted under the kidney capsule of immunodeficient mice (62). FGFs activate the ERK1/2 pathway to increase insulin mRNA (63). Of note, the contextual influence of islet ECM is demonstrated as this effect is modulated by integrin binding to laminin which causes a reduction in FGF receptor expression (64). Appraisal of native islet architecture warrants consideration in comparison to the post-isolation and early-engraftment islet state to appreciate the impact of the cell-cell and cell-matrix post-transplant microenvironment. Developing this area of knowledge specific to islet transplantation will open avenues to improve isolation, culture and engraftment in transplantation to promote survival and function (65).

## Islet isolation disrupts the microenvironment

### Revascularisation

During islet isolation, native vascularisation and surrounding matrix is destroyed. Significant collagen destruction (60-80%) is required to facilitate successful islet isolation (66). Consequently, islets are distributed into the portal venous system devoid of this supporting framework, increasing their vulnerability to apoptosis and necrosis whilst reliant on diffusion (67). Due to reliance on diffusion and despite placement into the highly vascularised liver site, this is insufficient to sustain islet populations. Cells within the central core of isolated islets demonstrate progressive reduction in viability when cultured *in vitro*, leading to cell death (68). Interestingly, smaller islets demonstrated improved responses to glucose stimuli *in vitro* and increased islet index (IEQ/islet number) positively correlates with stimulated C-peptide 3 months following simultaneous islet-kidney transplant (69). Hypoxia is a significant cause of islet failure in emerging encapsulation techniques and devices with smaller pore diameter are not favoured due to the prevention of revascularisation (70).

To achieve function after IT, revascularisation is a necessary requirement for successful islet engraftment. New intra-islet vasculature is derived from both recipient and donor endothelium (71, 72). Angiogenesis begins early after IT, likely from hepatic arterial tree (73) between day 1 and three and completes at approximately day 14 (74) with a following medium to long term period of vascular and extra-cellular matrix remodeling (67). In response to hypoxia, cultured and transplanted islets express VEGF, before an increase in VEGF receptor expression (Flk-1/KDR & Flt-1) between day 3-5 with a similar later rise in VE-Cadherin; these signs of revascularisation are reduced in diabetic small animal models compared to normoglycaemic controls, suggesting that a hyperglycaemic environment is unfavourable for islet survival (75).

Morphological analysis of autopsy specimens in long term functioning islet transplants (>10 years) with positive serum C-peptide has revealed extensive capillary networks positively staining for CD31, with comparable vascular density, size of vessel and distribution to those seen on histological analysis of biopsy tissue from native pancreas (76). Elsewhere, regardless of implantation site, post-transplant intra-islet vascular density is reduced compared to the native pancreas with oxygen tensions, compared to the highly arterialized supply of the native pancreas (77). The mechanisms of revascularisation and neo-angiogenesis may alter between transplant sites.

### Transplanted islets & extra-cellular matrix

**Figure 1:**
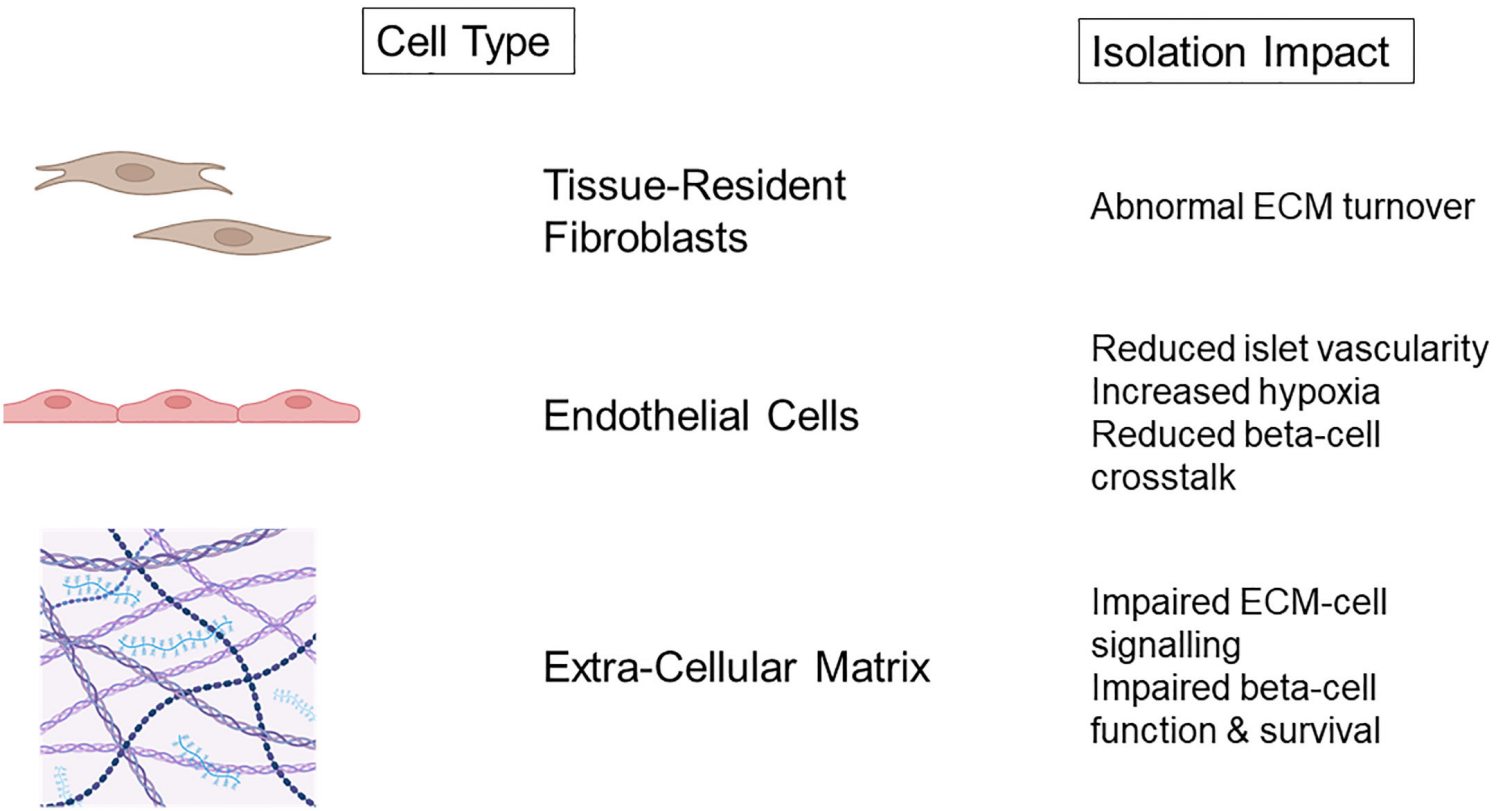
Non-endocrine cell damage and consequences of islet isolation. Created with BioRender.com.

To achieve successful islet isolation, destruction of the supporting framework of extra-cellular matrix vasculature is required (**Figure 1**) (66). Collagenase digestion non-selectively targets ECM components which breakdown peripheral pancreatic and intra-islet matrix which is associated with cell death (78). It is logical that reinstating the cell-matrix interactions following IT would be beneficial for islet survival and function.

Native pancreatic ECM is composed of both interstitial matrix and basement membrane, which is predominantly laminin, fibronectin alongside collagen IV and V (79). Islet isolation with collagenase targets both the interstitial matrix and BM and both are shown to be lost immediately after isolation of human and canine islets (79–81). Loss of the islet BM in native islets of non-obese diabetic mice has been correlated with increased macrophage infiltration and development of insulinitis, suggesting that these initially BM devoid islets post-transplant have increased vulnerability (82). Integrin expression is also reduced progressively during periods of islet culture (78), but this reduction is diminished when islets are cultured with fibronectin or collagen. Furthermore, the addition of fibronectin and collagen significantly reduced apoptosis during islet culture demonstrating the benefits of maintaining islet-ECM interactions (79, 80, 83). Insulin expression reduces in intensity during culture of human islets parallel to simultaneous reduction in integrin expression (79). Recently, a human decellularized pancreatic ECM model has been used to culture isolated human islets. This method has resulted in improvement of *in vitro* insulin dynamics, with superior glucose stimulated insulin secretion and reduced apoptosis in extended culture. Transcriptomic analysis demonstrated increased expression of regulatory pathways associated with cell adhesion, ECM organisation and responses to external stimuli when compared to culture in suspension (84).

Avenues to improve engraftment include increasing selectivity of collagenase digestion, promoting neovascularisation through manipulation of islets, co-culture with ECM components, non-endocrine cells and promoting islet-ECM interaction post-transplant should be investigated to improve cellular therapies (65, 85).

## Hepatic injury response & islet transplantation

### Islet loss after portal venous transplantation

The liver has many metabolic, digestive, and endocrine functions with complex micro-anatomical arrangement. The hepatic parenchyma receives dual blood supply from the portal venous system and hepatic arterial tree. Histologically, structures are arranged surrounding portal triads of portal venous sinusoids, arterial capillaries and bile duct branches with corresponding central veins which form the vascular outflow tract.

Following IT via the portal vein in man and primate models, islets will be spread amongst the liver with the majority sited within the venous lumen or closely related to it whilst only a minority become embedded within hepatic parenchyma (40, 76). Whereas, in small animal models, islets are most often seen within hepatic parenchyma, possibly due to endothelial breakdown as islets occlude the lumen in comparatively smaller vessels (76).

**Figure 2:**
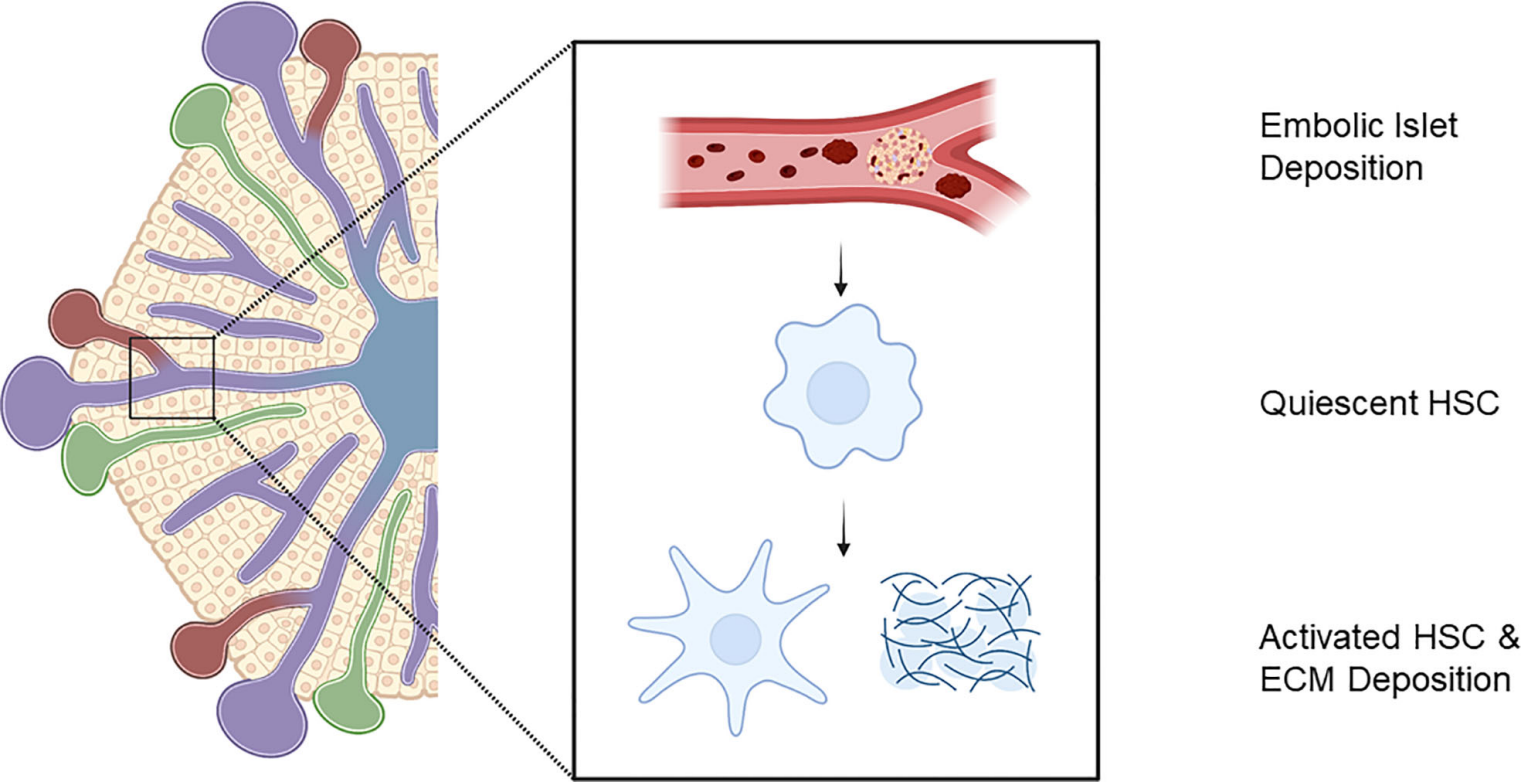
Embolic islet deposition within portal venous tree initiates liver injury response with activation of hepatic stellate cells and extra-cellular matrix deposition. Created with BioRender.com.

Intra-portal IT initiates the instant blood mediated immune response and triggers a response from the hepatic parenchyma (45). Embolic islets within the portal system result in areas of ischaemia inducing macroscopic changes in perfusion of the hepatic parenchyma in rodents and elevated hepatic enzymes in man (45). Hepatic stellate cells (HSC) located within the space of Disse in their quiescent state are responsible for ECM turnover and maintenance of the hepatic parenchymal environment (86). In response to injury, they undergo transformation into a myofibroblast that secretes tissue damaging ECM proteins (**Figure 2**). In chronic liver disease, such as non-alcoholic fatty liver disease, activation of HSCs is responsible for the pathological deposition of excessive matrix proteins associated with fibrosis, and the loss of conventional tissue architecture and function. Moreover, HSC activity is correlated with progressive liver disease and severity (87–89).

HSC activation in progressive fibrosis is detrimental to tissue function, their presence and matrix deposition surrounding intra-portally transplanted islets may influence islet survival and function. This local microfibrosis may interfere with diffusion of insulin, increasing distances to capillary perfusion and paracrine activity and after islet isolation, which destroys native islet ECM, this post-transplant matrix deposition may result in abnormal matrix-cell signalling, which may contribute to impaired beta-cell function and survival. Of note, streptozotocin induced hyerglycaemic mice fed a high fat diet to induce steatohepatitis, have significantly lower rates of euglycaemia after intra-portal IT with significantly higher non-fasting capillary glucose readings compared to controls (90).

It may be that in some scenarios, HSC induced matrix deposition helps to restore the islet-ECM signalling necessary for function. The nature of this consequent post-transplant matrix deposition needs to be investigated, as when isolated HSCs are co-transplanted with islets under the mouse kidney capsule, improved glycaemic control is demonstrated through increased duration of graft function and superior islet dynamics assessed with intra-peritoneal glucose tolerance testing. Histological analysis reveals HSCs are sited surrounding islet clusters in this setting, which was also associated with increased islet insulin expression on immunofluorescent staining compared to solitary islet transplant controls (91). In mice, this pattern of fibrosis surrounding islet grafts has been associated with long term functioning portal venous grafts at 200 days post-transplant, whereas, fibrosis detecting through Masson’ trichrome staining within islet grafts has been seen in graft failure; a pattern which can be reduced with pre-treatment with nicotinamide (92). HSCs have also been demonstrated to have a positive immune tolerance role after subcapsular renal transplantation, which may improve islet survival from immune mediated cell death (93, 94).

Transplanted islets may also influence their microenvironment through paracrine activity. Local hepatic histological changes have been noted in an early cohort of IT recipients and focal peri-portal steatosis has been demonstrated by magnetic resonance imaging and on histology (40, 95). Presence of focal steatosis was associated with higher requirements of exogenous insulin. Interestingly, amongst the insulin independent recipients in this cohort, those with hepatic steatosis also had higher serum C-peptide levels, suggesting that better functioning grafts produced more steatosis through paracrine action. In one case, steatosis resolved after graft failure, supporting a paracrine insulin mechanism (95). Of note, markers of HSC activation, including expression of aSMA, fibronectin and a1 procollagen are increased when isolated rat HSCs are treated after 3-4 passages *in vitro* with a combination of insulin and 15 mM glucose for 24 hours (96). In addition, insulin and IGF-1 have been shown to increase HSC proliferation *in vitro*; culture with IGF-1 also promoted collagen-1 accumulation in media (97). This demonstrates the validity of investigating how the two-way relationship between transplanted islets and the hepatic parenchyma influences this post-transplant microenvironment.

## Tissue response and alternative implantation sites

Alternative sites for IT away from the hostile hepatic parenchyma have been studied, with few being used outside of clinical trials with portal venous infusion continuing to be the imperfect gold standard (39, 98). Investigators have sought locations with high vascularity such as the spleen and the immune privileged thymus or testis to reduce allograft rejection as well as alternative non-invasive implantation sites such as subcutaneous fat and abdominal wall musculature. Non-invasive sites, which allow retrievability, are of interest with the emergence of encapsulation and cellular scaffolds which could be used in combination with islets of stem cell derived beta-cells (98). The local tissue response and microenvironment is likely to be as important in this setting as in the hepatic parenchyma.

The complexity of the relationship between the local tissue response and transplanted islets persists away from the hepatic parenchyma. When human and mouse islets are infused into the anterior eye chamber of syngeneic or immunodeficient mice, the islet BM which is destroyed during isolation is progressively reconstituted (99). Islets are encapsulated through a population of host fibroblasts which express vimentin, a marker of mesenchymal origin, pericyte marker PDGFRβ and PDGFRα, the fibroblast marker, but not alpha-smooth muscle actin, expression of which is a characteristic of myofibroblasts (99).

Subcutaneous tissue responses have been recruited against foreign bodies to create a collagen reaction surrounding an angiogram catheter, upon which islets can be placed. Islets can then be placed into the void created when the device is retrieved. This technique improves glycaemic control and graft survival in mouse syngeneic and allogeneic transplant models and when human islets are transplanted into an immunodeficient mouse model. Islet grafts are surrounded by a collagen capsule with evidence of neovascularisation within the graft (100). This provides further evidence of the supportive role of recipient fibroblast response and matrix deposition (100–102). Of note, the composition of this surrounding matrix deposition has not been characterised. Supplementation of ECM products to promote engraftment has also been trialled with success (103). Implantation of murine, porcine and human islets into the subcutaneous space of immunodeficient mice in combination with an islet viability matrix of collagen-1, L-glutamine, foetal bovine serum, sodium bicarbonate and medium 199 has shown improvements in both capillary glucose readings and human C-peptide production alongside maintenance of islet architecture on immunohistochemistry of explanted grafts (103).

More recently, immature pancreatic endocrine progenitors within an encapsulation device have been sited within the subcutaneous space with some success. Retrieved grafts have stained positive for insulin producing cells and recipients have had detectable levels of serum C-peptide. Histological analysis of explanted devices has demonstrated an influx of fibroblasts stained for alpha-smooth muscle actin, although quantitative analysis of this infiltrate relative to function has not been established; in areas of dense fibroblast infiltrate, there was minimal engraftment (17, 18). The role of this infiltrate warrants evaluation. The consequent matrix deposition may impair cell activity and progenitor differentiation to mature insulin producing cells.

## Summary

IT has the potential to provide insulin independence and reduction in mortality and morbidity induced by T1DM. But despite improvements in islet isolation techniques and immunosuppressive regimes, outcomes remain suboptimal. To improve duration and quality of glycaemic control after IT, it is important to examine the post-transplant microenvironment to understand the two-way relationship between transplanted islet cells and the hepatic parenchyma via paracrine and direct cell-to-cell and cell-ECM signalling. This will allow targeted improvements in islet isolation techniques and shed light on mechanisms for manipulation of the implantation microenvironment. This is an important area for improvements in both IT and stem-cell derived beta-cell therapies, for the benefit of patients living with DM.

## Author contributions

The article concept was produced by DTD, HAK, DvD, REJ, and KPH. The initial version was produced by DTD. All authors contributed to the article and approved the submitted version.
